# New York’s Board of Examiners

**Published:** 1895-10

**Authors:** 


### NEW YORK'S BOARD OF EXAMINERS.

IN answer to an editorial in the *American Veterinary Review* for August, we published in our September number an editorial which stated the facts in regard to the nomination and appointment of the New York State Board of Veterinary Medical Examiners. There appeared, however, in the September number of the *Review*, another editorial and a letter from Professor Law, which partly answers it, but the petty whining misstatements of this editorial seem to call for some further facts so that its readers shall not be misled. The *Review* talks of the action of the New York State Society as a "blunder" and not a thoughtless mistake, and refers to "strong opposition" and to a meeting of "eleven members," etc.

Every member of the New York State Society was duly notified of the meeting, and it can be assumed that those who take an active interest in the Society came. Those who have given days of work in the past attended and nominated men whom they knew were willing to sacrifice time to the interests of the Society.

Does the *Review* suppose that they would go into the by-ways and hunt up nominees in the "strong opponent" who had never attended a meeting of the Society nor paid his dues for three years, and in the faculty of the *Review's* college scarcely one of whom had ever taken the trouble to join the Society and help it in its work?

The *Review* refers to members of the Board interested in "certain schools." Dr. Law's letter in the September *Review* shows that he cannot possibly be interested in any graduates, as his prospective school will not graduate students before the year 1900; therefore, this can only refer to Dr. Huidekoper, recently connected with the New York College of Veterinary Surgeons.

The writer of the editorial in the *Review* can hold any opinion of Dr. Huidekoper's ability which he pleases to have, but he would scarcely dare tell Dr. Huidekoper that he questions his honesty in examinations. Would the editor of the *Review* like to publish the examination papers which Dr. Huidekoper returned to the American Veterinary College, when he lectured there, and the list of graduates?

"And thus we" (do) "stand." "The law has been passed, "the State Society has furnished the Regents with the pre- "scribed ten names, and they have appointed the five *disinter-* "*ested* veterinarians, who are to hold office as examiners."

"That disgraceful special meeting," which is a "blot upon the good name of the State Society," was composed of the men who elected the editor of the *Review* an "Honorary Member" of the Society some years ago, and they have never had the courtesy of an attendance from him or his coterie of veterinarians, the labor of a days' work in legislation from them, nor the subscription of a dollar in its interest from them.

"Cat's-paws," "glacér climbers" who slide back 999 feet more than each step they advance, and "financial benefits" of a teacher in a veterinary institution, is gallery twaddle. When the *American Veterinary Review* will take the trouble and expense to send a fair representative to the State Society's meeting and enter into honest debate, it may be competent to discuss the Society's actions; until then it had better not depend upon information furnished to it by cranks.

